# Comparative transcriptomic analysis of salivary glands between the zoophytophagous *Cyrtorhinus lividipennis* and the phytozoophagous *Apolygus lucorum*

**DOI:** 10.1186/s12864-023-09956-4

**Published:** 2024-01-11

**Authors:** Fang He, Yang-Wei Gao, Zhuang-Xin Ye, Hai-Jian Huang, Cai-Hong Tian, Chuan-Xi Zhang, Jian-Ping Chen, Jun-Min Li, Jia-Bao Lu

**Affiliations:** 1 State Key Laboratory for Managing Biotic and Chemical Threats to the Quality and Safety of Agro-products, Key Laboratory of Biotechnology in Plant Protection of MARA and Zhejiang Province, Institute of Plant Virology, Ningbo University, 315211 Ningbo, China; 2 Institute of Plant Protection, Henan Academy of Agricultural Sciences, 450002 Zhengzhou, China; 3 Institute of Insect Science, Zhejiang University, 310058 Hangzhou, China

**Keywords:** Saliva, Salivary gland, Transcriptome, Genome, *Cyrtorhinus lividipennis*, *Apolygus lucorum*

## Abstract

**Background:**

Saliva plays a crucial role in shaping the feeding behavior of insects, involving processes such as food digestion and the regulation of interactions between insects and their hosts. *Cyrtorhinus lividipennis* serves as a predominant natural enemy of rice pests, while *Apolygus lucorum*, exhibiting phytozoophagous feeding behavior, is a destructive agricultural pest. In this study, a comparative transcriptome analysis, incorporating the published genomes of *C.lividipennis* and *A.lucorum*, was conducted to reveal the role of salivary secretion in host adaptation.

**Results:**

In contrast to *A.lucorum*, *C.lividipennis* is a zoophytophagous insect. A *de novo* genome analysis of *C.lividipennis* yielded 19,706 unigenes, including 16,217 annotated ones. On the other hand, *A.lucorum* had altogether 20,111 annotated genes, as obtained from the published official gene set (20,353 unigenes). Functional analysis of the top 1,000 salivary gland (SG)-abundant genes in both insects revealed that the SG was a dynamically active tissue engaged in protein synthesis and secretion. Predictions of other tissues and signal peptides were compared. As a result, 94 and 157 salivary proteins were identified in *C.lividipennis* and *A.lucorum*, respectively, and were categorized into 68 and 81 orthogroups. Among them, 26 orthogroups were shared, potentially playing common roles in digestion and detoxification, including several venom serine proteases. Furthermore, 42 and 55 orthogroups were exclusive in *C.lividipennis* and *A.lucorum*, respectively, which were exemplified by a hyaluronidase in *C.lividipennis* that was associated with predation, while polygalacturonases in *A.lucorum* were involved in mesophyll-feeding patterns.

**Conclusions:**

Findings in this study provide a comprehensive insight into saliva secretions in *C.lividipennis* and *A.lucorum* via a transcriptome approach, reflecting the intricate connections between saliva secretions and feeding behaviors. It is found that conserved salivary secretions are involved in shaping the overlapping feeding patterns, while a plethora of unique salivary secretions may drive the evolution of specific feeding behaviors crucial for their survival. These results enhance our understanding of the feeding mechanisms in different insects from the perspective of saliva and contribute to future environmentally friendly pest control by utilizing predatory insects.

**Supplementary Information:**

The online version contains supplementary material available at 10.1186/s12864-023-09956-4.

## Background

Saliva plays diverse roles in the process of insect feeding, ranging from the initiation of penetration to the regulation of host defenses [1–3]. For insects equipped with piercing-sucking mouthparts, saliva is firstly secreted into plant tissues or other prey during feeding. Abundant digestive enzymes and hydrolases are contained in the saliva for extra-oral digestion, including prevalent serine protease [3–6]. In addition, several enzymes and non-enzymatic proteins in saliva exert important effects on regulating the intricate interactions between insects and hosts. Hosts can employ diverse defense strategies in response to insect feeding, such as the synthesis of secondary metabolites and other adverse factors. In turn, insects evolve further counter-defense strategies to overcome the host defenses and acquire nutrition [7–9]. Given the vast array of hosts and diverse feeding behaviors exhibited by insects, the salivary approach will shed more light on the evolutionary adaptation of insects to their hosts. Salivary glands (SGs), which are the primary tissue for saliva secretion, have attracted increasing attention. The prediction of salivary secretions through SGs transcriptome analysis is a prevalent approach in numerous insects, including planthopper and plant bug [10–12].

In the order Hemiptera, the family Miridae stands out as one of the most species-rich groups, which is characterized by a broad feeding range with piercing-sucking mouthparts [13, 14]. The feeding habits of mirid bugs are notably intricate, spanning from strict phytophagous to carnivorous insects [14]. In addition, the polyphagous type is also prevalent in the family Miridae, presenting as facultative animal feeding in conjunction with phytophagous habits (phytozoophagous) or vice versa (zoophytophagous), as exemplified by species such as *Dicyphus tamaninii*, *Lygus Hesperus*, and *Lygus lineolaris* [15, 16]. To date, extensive investigations have been conducted to elucidate the impacts of diverse diets on the biological parameters of mirid bugs, make use of predominant carnivorous insects as biological control agents, and clarify the ecological niches of phytophagous pests in the context of pest management programs [17, 18]. For instance, in the rice ecosystem, *Tytthus chinensis* may probably serve as an effective biological control agent under future global warming conditions [19]. Conversely, *Stenotus rubrovittatus*, a rice pest, may benefit from the presence of a non-native plant, *Lolium multiflorum*, before it colonized rice fields [20]. However, the intricate mechanisms underlying the complex feeding habit remain largely unclear. To better understand the adaptive strategies employed by mirid bugs in their feeding behaviors, it will be an effective approach to comprehensively investigate the SGs.

*Cyrtorhinus lividipennis* (Hemiptera, Miridae) is a predominant natural enemy in rice fields, preying on eggs and nymphs of rice pests like planthoppers and leafhoppers [21]. Notably, *C.lividipennis* employs a plethora of odorant-binding proteins and chemosensory proteins to efficiently identify insect eggs embedded in the leaf sheath of rice [22]. This provides *C.lividipennis* with a distinct advantage in suppressing the outbreak of rice pests featured by high fertility. In contrast, *Apolygus lucorum* (Hemiptera, Miridae), a destructive agricultural pest, is widely distributed in Asia, Africa, America, and Europe, and displays a broad spectrum of host plants, especially cotton [23]. *A.lucorum* primarily sucks the sap of the tender leaves, buds, flowers, fruits, and anthers of host plants, additionally, it also preys on small insects and insect eggs, like *Bemisia tabaci* and the egg of *Helicoverpa armigera* [24], highlighting its phytozoophagous trait. Recently, the genomes of both *C.lividipennis* [25] and *A.lucorum* [26] have been reported, making these two mirid bugs the ideal models for investigating salivary secretions during feeding.

In this study, we first observed and recorded the impact of rice seedlings alone on the survival of *C.lividipennis*, the natural enemy of rice pests. Subsequently, a comprehensive comparative analysis of the top 1000 highly expressed genes (top 1000 genes) in SGs of *C.lividipennis* and *A.lucorum* was conducted through transcriptomic profiling of the SGs. Further, comparative analysis with other tissues was performed to screen out the significantly highly expressed genes in these top 1000 genes in SGs and the accuracy was validated by quantitative real-time polymerase chain reaction (qRT-PCR). Thereafter, the putative salivary secretions in *C.lividipennis* and *A.lucorum* were obtained by predicting signal peptides. Ultimately, a comparative analysis of the putative salivary secretions from these two mirid bugs was performed to unveil the similarities and differences. In addition, the expression level of several important salivary secretion-coding genes was studied by qRT-PCR to reveal their potential roles during feeding. This comprehensive analysis in this study can shed light on the underlying mechanisms behind the various feeding behaviors of *C.lividipennis* and *A.lucorum*, thereby laying a valuable theoretical foundation for the development of effective pest management strategies by harnessing predatory insects.

## Results

### Feeding behavior of *C.lividipennis*

**Fig. 1:**
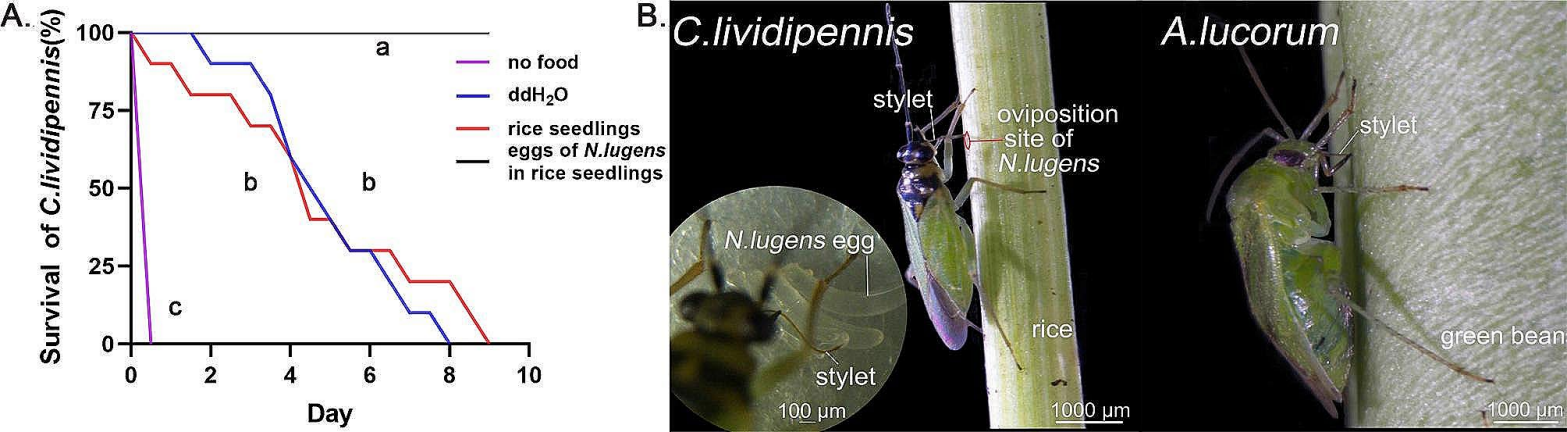
Feeding behaviors of *C.lividipennis* and *A.lucorum*. **A.** Survival of *C.lividipennis* feeding on rice seedlings and eggs of *N.lug*ens in rice seedlings, ddH_2_O feeding and no feeding were the control groups. **a**, **b**, **c**: Different letters indicate significant differences between two groups (*P* < 0.05); n = 10, for each treatment. **B.** Left panel: *C.lividipennis* feeding on eggs of *N.lugens* in rice seedlings; lower left panel: *C.lividipennis* feeding on the dissected eggs of *N.lugens*; Right panel: *A.lucorum* feeding on green beans

In the process of feeding on insect eggs inside rice tissue, the stylet of *C.lividipennis* initially penetrated the rice tissue and later gained access to insect eggs, like *N.lugens* eggs. A single food supply experiment was performed to elucidate the impact of rice seedlings on *C.lividipennis* (Fig. 1A). As revealed by survival analysis, *C.lividipennis* utilized rice seedling sap as a supplementary liquid source, but animal food was necessary for its prolonged survival. The above result verified the zoophytophagous trait of *C.lividipennis*, which was distinct from the phytozoophagous *A.lucorum* (Fig. 1B) [18, 23].

### Transcriptome mapping and gene annotation of *C.lividipennis* and *A.lucorum*

Using the Illumina Novaseq 6000 platform, RNA-sequencing (RNA-seq) libraries of the SGs of both *C.lividipennis* and *A.lucorum* were constructed. Based on the genomes of *C.lividipennis*, the Braker tool was employed to predict gene structures via a *de novo* prediction method, which generated altogether19706 unigenes. As for *A.lucorum*, a total of 20,353 predicted unigenes from a prior study [26] were employed for subsequent analyses. Then, all unigenes were searched against the NCBI NR, Uniprot, and Gene Ontology (GO) databases, finally yielding 16,217 and 20,111 annotated unigenes for *C.lividipennis* and *A.lucorum*, respectively (Supplementary Table 1A, B).

### Function analyses of SGs-abundant genes in *C.lividipennis* and *A.lucorum*

To comprehensively assess the functions of SGs, the top 1000 SG-abundant genes were analyzed (Supplementary Table 2A, B). Notably, ribosomal proteins accounted for the highest proportion in both insects, with 81 and 71 being identified in *C.lividipenni*s and *A.lucorum*, respectively. Additionally, substantial proportions of genes-18.8% in *C.lividipenni*s and 21.9% in *A.lucorum*-were predicted to contain a signal peptide. This suggests active protein synthesis and secretion processes through the endoplasmic reticulum membrane pathway in SGs [27]. Besides, genes identified in several insects were highly expressed in the SGs of both mirid bugs, including carbonic anhydrase, carboxypeptidase, and calcium-binding proteins (Calreticulin, Aralar1, and annexin). Additionally, there were 44 *C.lividipennis*-specific and 32 *A.lucorum*-specific genes identified, with no homologs being discovered from eight other insect databases.

**Fig. 2:**
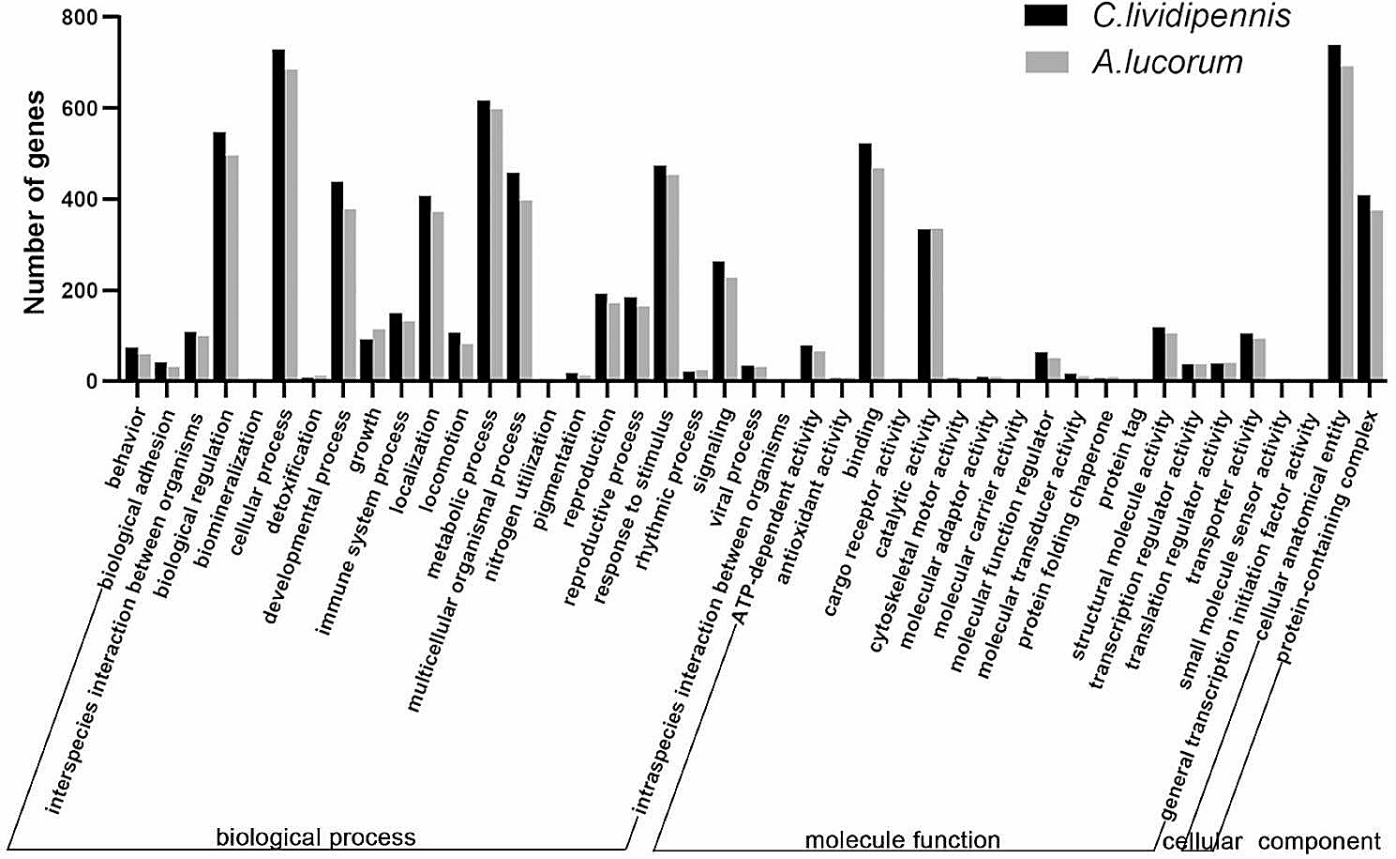
GO analysis of the top 1000 salivary gland-abundant genes in *C.lividipennis* and *A.lucorum*

Subsequently, GO annotations of the top 1000 genes in the SGs were compared between the two insects. As a result, altogether 43 and 40 function sets were conclusively classified in *C.lividipennis* and *A.lucorum*, respectively (Fig. 2, Supplementary Table 3A, B). Notably, GO annotations exhibited striking similarities in both species. In the biological process category, most genes were enriched into the cellular process, metabolic process, and biological regulation; with regard to the molecular function category, most genes were associated with binding and catalytic activity; while, only two sub-terms were annotated in the cellular component category, including the cellular anatomical entity and protein-containing complex. Collectively, GO annotations unveil the SGs as the dynamically active tissues engaged in protein synthesis and secretion, underscoring their pivotal role in the feeding processes of both *C.lividipennis* and *A.lucorum*.

### Tissue expression analyses in *C.lividipennis* and *A.lucorum*

**Fig. 3:**
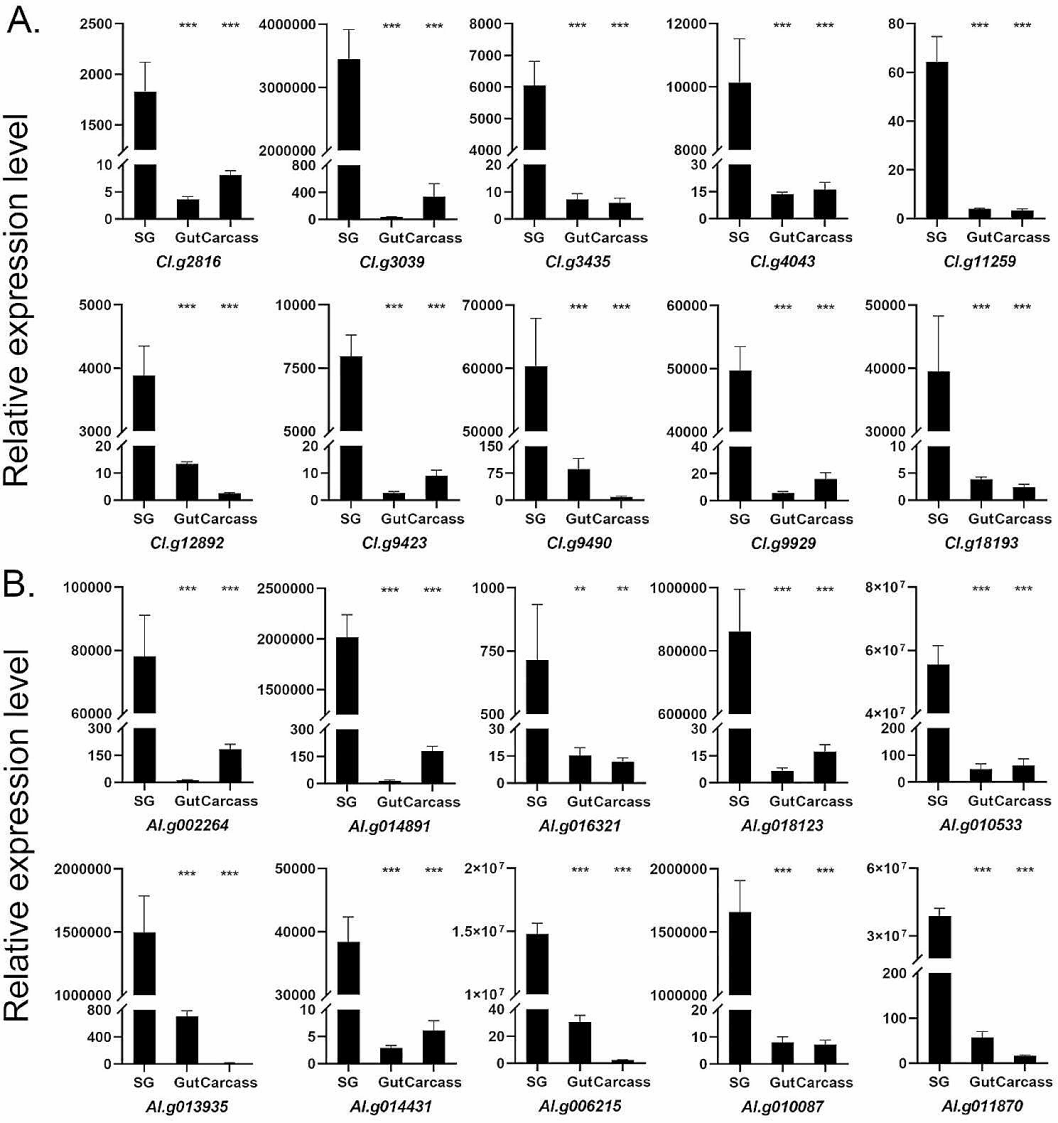
Tissue expression profiles of 20 genes are verified by qRT-PCR for *C.lividipennis* (**A**) and *A.lucorum* (**B**)

In comparison with the transcriptomes of the gut and carcasses, 144 genes in *C.lividipennis* and 256 genes in *A.lucorum* were significantly highly expressed in SGs than in the other two tissues (Supplementary Table 2A, B). To further validate these findings, 20 genes were selected based on annotation and signal prediction for qRT-PCR analysis. As a result, these genes were markedly up-regulated in the SGs of both insect species, consistent with the transcriptome analysis results (Fig. 3).

### Identification of salivary proteins in *C.lividipennis* and *A.lucorum*

**Fig. 4:**
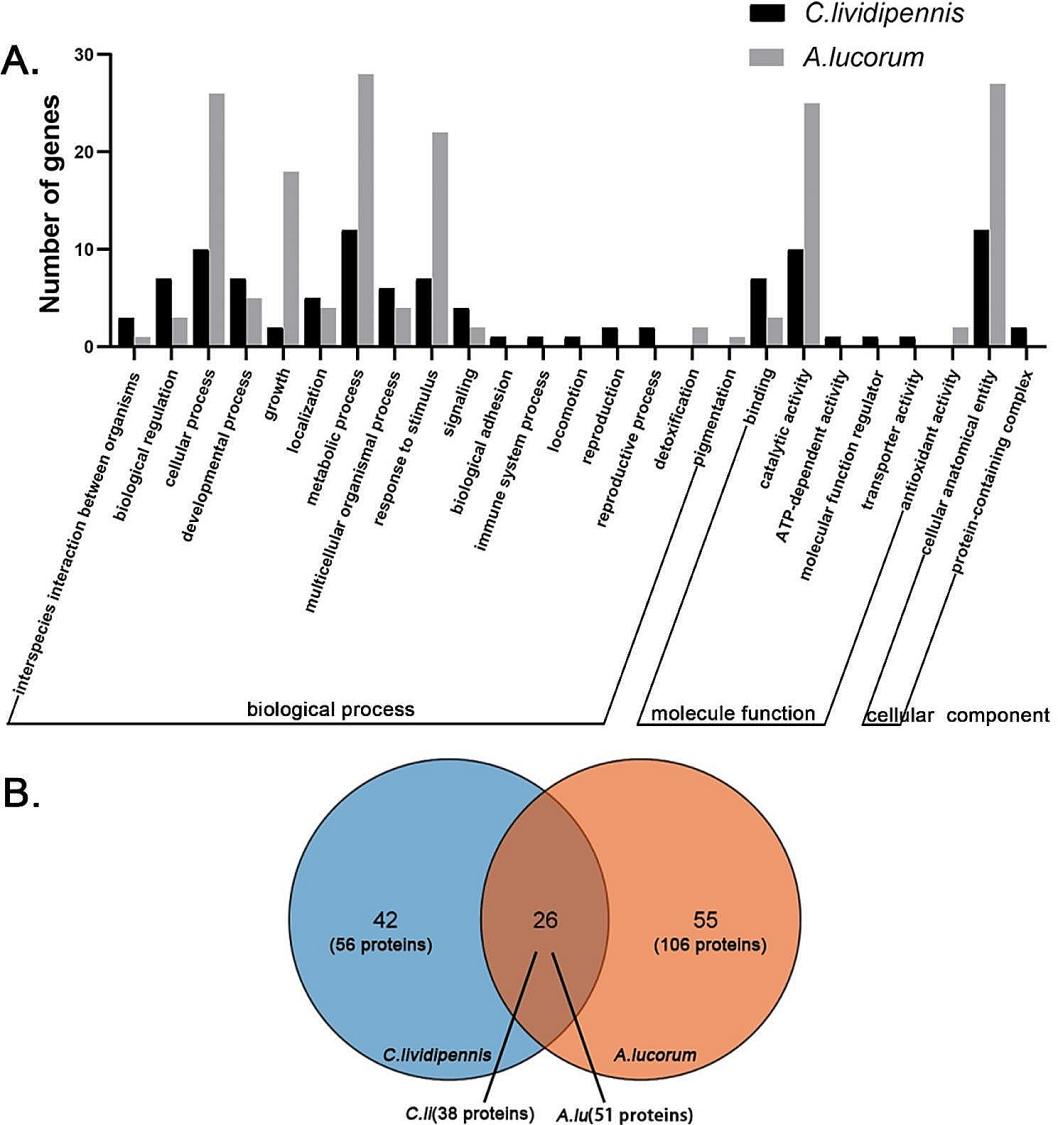
GO analysis of the putative salivary proteins (**A**) and venn diagram of the putative salivary proteins orthogroups (**B**) in *C.lividipennis* and *A.lucorumC.li*: *C.lividipennis*; *A.lu*: *A.lucorum*

The abundantly and significantly highly expressed proteins with secreted characteristics in the SG transcriptome were identified as the putative salivary secretions, and a total of 94 putative salivary secretions with an N-terminal signal peptide were identified in *C.lividipennis* (Table 1, Supplementary Table 4A). According to GO analysis, the majority of these salivary secretions were associated with metabolic process (12 proteins), cellular anatomical entity (12 proteins), cellular process (10 proteins), and catalytic activity (10 proteins) (Fig. 4A, Supplementary Table 3A). Notably, some proteins, including venom serine protease, chymotrypsinogen B2, pancreatic lipase-related protein 2, venom nuclease 1, and hyaluronidase, were involved in hydrolysis. Additionally, peptidase M14 carboxypeptidase A domain-containing protein exhibited peptidase activity. Besides, a nucleobindin-2 isoform X2, which is an EF-hand domain-containing protein, participated in calcium binding [28]. Moreover, proteins such as tRNA pseudouridine synthase A and polypeptide N-acetylgalactosaminyltransferase 3 might be implicated in translation and posttranslational modification, respectively. Notably, a salivary-secreted peptide was identified. Furthermore, proteins including laccase-2 isoform A, serpins-containing protein (Ovalbumin-related protein Y), salivary lipocalin, and C/SUEL-type lectin domain-containing protein were responsible for immune resistance.

**Table 1:** Putative salivary secretions in *C.lividipennis* and *A.lucorum*

Functional category of proteins	Description	Number in *C.lividipennis*	Number in *A.lucorum*
Digestion	Venom serine protease	10	10
	Muramidase	1	1
	Venom nuclease 1	1	1
	Trypsin	2	2
	pancreatic lipase-related protein	1	1
	Endonuclease	1	1
	Hyaluronidase	1	0
	Lipase domain-containing protein	1	0
	Chymotrypsinogen B2	1	0
	Secreted venom protein family 3 protein	1	0
	Polygalacturonase	0	7
	Endopolygalacturonase	0	11
	Trehalase	0	1
Immune related	Laccase-2 isoform A	1	1
	Protease inhibitor	1	1
	Nucleobindin-2	1	1
	serine threonine-protein kinase	1	0
	Cathepsin D	1	0
	Chondroitin proteoglycan-2	1	0
	SUEL-type lectin domain-containing protein (Fragment)	1	0
	Polypeptide N-acetylgalactosaminyltransferase 3	1	0
	Salivary lipocalin	1	0
	Ovalbumin-related protein Y	1	0
	tRNA pseudouridine synthase A	1	0
	Nose resistant-to-fluoxetine protein N-terminal domain-containing protein	1	0
	C-type lectin domain-containing protein	1	0
	Proclotting enzyme	1	0
	Mucin-like domain-containing protein	0	1
	Cystatin domain-containing protein (cysteine protease inhibitors)	0	1
	Pathogenesis-related protein	0	2
	Kazal-like domain-containing protein (Kazal type serine protease inhibitors)	0	1
	Pappalysin-1 (insect immune response protein)	0	1
	Basic proline-rich protein-like (Collagens are generally extracellular structural proteins involved in formation of connective tissue structure.)	0	1
	Glutathione S-transferase	0	2
	Apolipophorin-III	0	3
	Cuticular protein RR-2 motif	0	1
	Chaperone protein DnaJ	1	0
	Larval cuticle protein A2B	0	1
Peptide related	carboxypeptidase	2	2
	salivary secreted peptide	1	4
Binding	ATP-binding cassette sub-family G member 1	1	0
	Lipid-binding serum glycoprotein N-terminal domain-containing protein	0	3
Others	Putative translation initiation factor IF-2	1	1
	Valine-tRNA ligase	0	1
	RNA polymerase-associated protein RapA (Fragment)	0	1
	Nucleoprotein TPR	0	1
Unknown function	-	54	92

In total, 157 putative salivary secretions were predicted in *A.lucorum* (Table 1, Supplementary Table 4B). As unveiled by GO analysis, proteins associated with metabolic process (28 proteins), cellular anatomical entity (27 proteins), cellular process (26 proteins), and catalytic activity (25 proteins) played dominant roles (Fig. 4A, Supplementary Table 3B). Among these 157 putative salivary secretions, 7 polygalacturonases and 11 endo-polygalacturonases exerted the major effects, indicating a robust ability to digest plant foods. Additionally, venom serine protease and trehalase were implicated in hydrolysis. Similar to *C.lividipennis*, a peptidase M14 carboxypeptidase exhibited peptidase activity and a nucleobindin-2 isoform X1 participated in calcium binding. Besides, 4 other salivary-secreted peptides were identified. Other genes, including mucin-like domain-containing protein, Glutathione S-transferase (GST), laccase-2 isoform A, and apolipophorin-III participated in immune resistance. Notably, there were altogether 25 *C.lividipennis*-specific and 19 *A.lucorum*-specific salivary proteins identified ultimately. No homologs were detected from the databases of the other eight representative insects.

### Comparative analyses of the putative salivary proteins in *C.lividipennis* and *A.lucorum*

To elucidate the mechanisms underlying the distinct feeding behaviors via the salivary approach, a comparative analysis of the above putative salivary secretions in both insects was conducted using the OrthoFinder program. Therefore, the putative salivary proteins were further classified into 68 and 81 orthogroups with different functions in *C.lividipennis* and *A.lucorum*, respectively. Of them, 26 orthogroups overlapped, whereas 42 and 55 were exclusive to *C.lividipennis* and *A.lucorum*, separately (Fig. 4B, Supplementary Tables 5–7).

Among the 26 common orthogroups, several essential hydrolases fundamental for digestion were identified, including venom serine protease, venom nuclease 1, muramidase, and lipase domain-containing protein. In addition, multiple proteins were discovered from the detoxification processes, mainly including laccase-2 isoform A, nucleobindin-2 isoform X2, protease inhibitor, and cystatin domain-containing protein. A peptidase M14 carboxypeptidase A domain-containing protein, which is prevalent in various insects, like *Laodelphax striatellus* and *N.lugens*, exhibited peptidase activity [10]. The presence of translation initiation factor IF-2 suggested an active role in protein synthesis in the SGs. Additionally, 11 groups of unknown proteins were also identified.

Apart from the above common orthogroups discovered in *C.lividipennis* and *A.lucorum*, there were several proteins exclusively existing in each species. Notably, a prominent feature related to general digestion was the abundance of polygalacturonases in *A.lucorum*, indicating the active involvement in the degradation of plant cell walls. Moreover, 6 venom serine protease 34 and 1 venom serine protease were found in *A.lucorum*, whereas 9 distinct venom serine protease 34 existed in *C.lividipennis*. In addition, there were several distinctive proteins implicated in overcoming plant resistance. In *A.lucorum*, the GST, mucin-like domain-containing protein, apolipophorin-III, trehalase, Lipocalin/cytosolic fatty-acid binding domain-containing protein, and Kazal-like domain-containing protein were identified, which played roles in the breakdown of plant secondary metabolites and xenobiotics detoxification. Moreover, pappalysin-1, basic proline-rich protein-like, venom allergen 5, cuticle protein, and pathogenesis-related protein 5 were crucial for regulating the interaction between insects and plants in the feeding process. By contrast, in *C.lividipennis*, key proteins such as serine/threonine protein kinase, C/SUEL-type lectin domain-containing protein, cathepsin D, chondroitin proteoglycan-2, salivary lipocalin, and polypeptide N-acetylgalactosaminyltransferase 3 were involved in host adaption. Additionally, a hyaluronidase in *C.lividipennis* might facilitate the activities of other saliva digestion enzymes during predation. These results reflect the intricate strategies employed by *C.lividipennis* and *A.lucorum* during their feeding behaviors.

### Function analyses of 10 salivary proteins

**Fig. 5:**
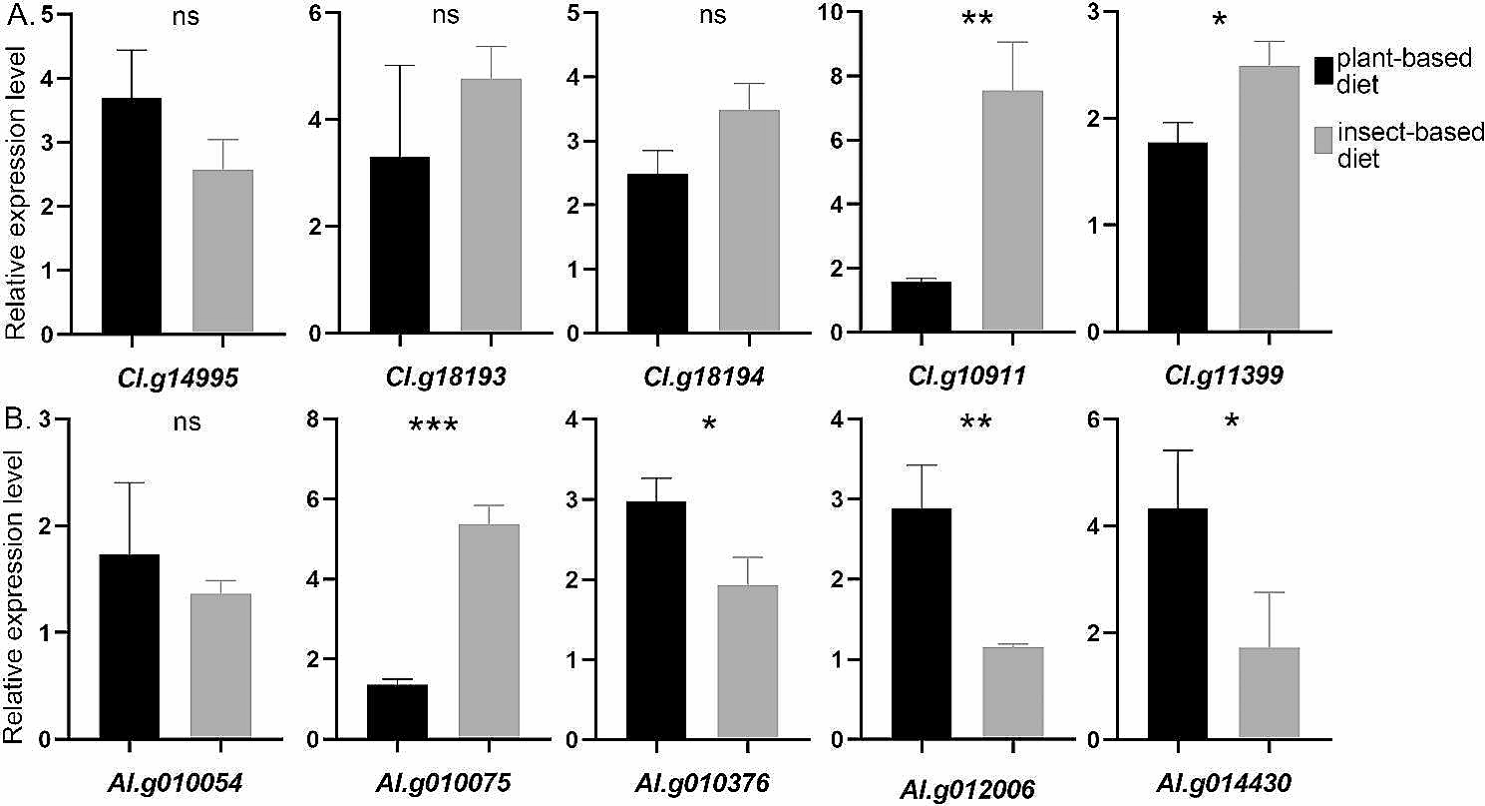
Expression profiles of 10 genes analyzed by qRT-PCR for *C.lividipennis* (A) and *A.lucorum* (B) with plant- or insect-based foods. For *C.lividipennis* individuals: rice seedlings were used as plant-based food, and eggs and nymphs of *N.lugens* were used as insect-based foods. For* A.lucorum*, green beans were used as plant-based food, and eggs of *H.armigera* were used as insect-based foods. Data are means + SEM, three biological replicates. ns: *P* > 0.05, indicating no significant difference; *P < 0.05, **P < 0.01, and ***P < 0.001, indicating significant, very significant, and extremely significant differences

After consuming insects or plants for 4 days, the expression levels of 10 salivary protein-coding genes were evaluated to elucidate their roles in the distinct feeding behaviors exhibited by *C.lividipennis* and *A.lucorum* (Fig. 5). A shared orthogroup (OG0000005) associated with general digestion was first selected, which consisted of 3 venom serine proteases in *C.lividipennis* and 2 in *A.lucorum*. Among them, 4 genes (*Cl.g14995*, *Cl.g18193*, *Cl.g18194*, *Al.g010054*) exhibited no difference in expression, while 1 (*Al.g010075*) was up-regulated with an insect-based diet. These findings indicate that the former 4 serine proteases perform basic hydrolysis irrespective of the type of diet (insect or plant), while the latter 1 serine protease shows a preference for digesting insect-based food. Besides, 5 genes that exist separately in one of these two species were studied. In *A.lucorum*, the up-regulation of a polygalacturonase (*Al.g012006*), crucial for the degradation of plant cell walls, was observed in response to a plant-based diet. Moreover, a GST (*Al.g014430*) and a Kazal-like domain-containing protein (*Al.g010376*) demonstrated up-regulation in response to a plant-based feeding, suggesting the potential contribution of these two proteins to resistance against plant defense mechanisms. In the case of *C.lividipennis*, the up-regulation of a hyaluronidase (*Cl.g11399*), important for predation, was noted in response to an insect-based diet. Furthermore, a SUEL-type lectin domain-containing protein (*Cl.g10911*) exhibited up-regulation during insect-based feeding, thereby indicating its involvement in the modulation of insect immunity.

## Discussion

Saliva plays a crucial role in shaping the feeding behavior of an insect and is involved in ex-oral digestion and host adaptation [29–31]. This study delved into the intricate feeding behaviors of *C.lividipennis* and *A.lucorum* from a saliva-centric perspective. Numerous conserved and unique saliva proteins were identified in these two insects through comparative analysis of the SGs transcriptomes. Findings in this study offer a comprehensive understanding of the salivary secretions, revealing how saliva contributes to the evolution of zoophytophagous and phytozoophagous feeding behaviors in *C.lividipennis* and *A.lucorum*.

### Salivary secretions associated with digestion

Among various hydrolases present in the insect saliva, serine protease stands out as one of the most widely distributed enzymes and is a multifunctional protein associated with various physiological processes, including digestion, immunity, and reproduction [6, 32–34]. In this study, 17 containing 10 venom serine proteases, 2 trypsins, 1 Chymotrypsinogen B2, 1 Proclotting enzyme, 3 hypothetical proteinsand 12 (containing 10 venom serine proteases and 2 trypsins) abundantly expressed serine protease-related proteins were identified in *C.lividipennis* and *A.lucorum*, respectively, highlighting the importance of saliva for the feeding process. Except for one serine protease (Cl.g14408), a typical serine protease domain (Tryp_SPc) and a His-Asp-Ser (H-D-S) catalytic triad were present in all the other enzymes, indicating their active digestive functions [35] (Supplementary Fig. 1). Among these enzymes, 9 in *C.lividipennis* and 5 in *A.lucorum* were classified into homologous groups, while 8 (including Cl.g14408) and 7 distinct enzymes were identified in *C.lividipennis* and *A.lucorum*, respectively. Besides, muramidase, venom nuclease 1, lipase domain-containing protein, and peptidase M14 carboxypeptidase A domain-containing protein were discovered in both insects. Muramidase was classified as an O-Glycosyl hydrolase, which possessed a Glyco_25 domain [36]. Peptidase M14 carboxypeptidase A domain-containing protein, a zinc-binding protein with protein-hydrolyzing function, was detected in a range of hemipterans [37]. Thus, it is reasonable that these two mirid bugs share fundamental hydrolysis functions.

*C.lividipennis*, as a predatory bug, shows a preference for *N.lugens* eggs that are laid in rice seedlings [38]. In addition to the above hydrolases, *C.lividipennis* exclusively possesses the hyaluronidase, a well-documented representative in the saliva of predators for extra-oral digestion [39]. Notably, hyaluronidase is commonly found in the venom of various predatory arthropods, such as assassin bugs [40] and spiders [41]. Previous studies have indicated that hyaluronidase serves as a “spreading factor”, which facilitates the spreading of toxins or saliva in prey tissues by breaking down the substances around cells [39]. A higher expression level of the hyaluronidase in *C.lividipennis* feeding on eggs of *H.armigera* supported its significance for predation. We speculate that the hyaluronidase in *C.lividipennis* functions to enhance the efficient infiltration of other salivary secretions, thereby accelerating the paralysis and digestion of prey tissues.

*A.lucorum* displays a mesophyll-feeding behavior [26], which is observed in various mirid bugs, including *L.lineolaris* [11]. Polygalacturonase, also known for its pectin degradation capability, is widely distributed in the saliva of phytophagous insects like aphids and leafhoppers. This enzyme can facilitate the penetration of plant tissues, thus promoting nutrient absorption [42, 43]. Previous studies have emphasized the role of polygalacturonase as a primary factor for inducing visible plant injury [44]. Among the salivary secretions of *A.lucorum*, a total of 22 polygalacturonase-related proteins (containing 7 polygalacturonases, 11 endopolygalacturonases, and 4 hypothetical proteins) were identified. It is hypothesized that polygalacturonase contributes to foraging on a broad range of plant types by breaking down plant cell walls and facilitating nutrient ingestion, akin to the process observed in *Lygus hesperus* [45]. As revealed by structural domain analysis (Supplementary Fig. 2), all the enzymes possess the typical structure of parallel beta-helix repeats, except for two (Al.g012038 and Al.g016675) that contain the Glyco_hydro_28 domains [46]. Notably, none of these polygalacturonases was identified in the putative salivary secretions of *C.lividipennis*, regardless of its initial penetration into rice tissue before feeding on insect eggs. This suggests that the plant-piercing mechanism employed by *C.lividipennis* is distinct from that of *A.lucorum*. Additionally, trehalase and carboxypeptidase were individually identified in the saliva of *A.lucorum*. Trehalase is a digestive enzyme responsible for hydrolyzing trehalose into glucose molecules, and it has been identified in the saliva of several phytophagous insects, including *Lygus rugulipennis* [47], *Sitobion avenae*, and *Metopolophium dirhodum* [48]. Besides, carboxypeptidase, an enzyme involved in digestion, has also been identified in another phytozoophagous insect, *L.lineolaris* [11]. In summary, the presence of different digestion-related enzymes reflects the evolution of unique digestion modes in response to distinct hosts.

### Salivary secretions associated with host adaption

Salivary secretions have been recognized with key roles in assisting insects in adapting to their hosts [29, 49]. Predictions of salivary secretions in both *C.lividipennis* and *A.lucorum* unveiled the presence of several proteins commonly regulating the insect-host interactions, including laccase-2 isoform A, nucleobindin-2 isoform X2, protease inhibitor, and cystatin domain-containing protein. Laccase, which promotes the oxidization of the potentially toxic monolignols into nontoxic polymers, has been implicated in various insect-feeding adaptations, mostly in herbivorous insects [50, 51]. Nucleobindin-2, a calcium-binding protein, counteracts the calcium-triggered sieve-tube occlusion, suggesting its potential role in stylet penetration [52]. For von Willebrand factor (vWF) type C domain–containing protease inhibitor, previous reports have shown that two homologs (CG15199 and CG15202) of this protease inhibitor are detected in the *Drosophila* embryonic SGs, and CG15199 is discovered with nutrition regulatory effect [53]. The above results imply that the facultative rice sap feeding pattern of *C.lividipennis* and the main plant feeding behavior of *A.lucorum* were correlated with these 3 enzymes. Furthermore, cystatin domain-containing protein, another type of protease inhibitor, has been studied in tick saliva, and is found to participate in the immune response during tick feeding [54]. The presence of the cystatin domain-containing protein indicates the facultative animal feeding of *A.lucorum* and the main animal feeding behavior of *C.lividipennis*.

In addition to the aforementioned shared enzymes, unique detoxifying enzymes or non-enzymic proteins were present in each of the two species, facilitating their respective predominant feeding processes. As for *C.lividipennis*, several proteins typically associated with animal-feeding insects were identified. In a previous study, the presence of a rhamnose-binding lectin and a substantial quantity of lipocalin family proteins were confirmed in the saliva of *Triatoma dimidiate* [55]. In our study, two galactoside-binding lectins (C/SUEL-type lectin domain-containing proteins) and a lipocalin protein were discovered in *C.lividipennis*. These 2 galactoside-binding lectins act as the pattern-recognition receptors and are responsible for the insect innate immune system of the insect [56, 57]. Notably, a higher expression level of the SUEL-type lectin domain-containing protein was induced when feeding on insect foods, verifying the above speculation. A homolog of Ovalbumin-related protein Y (serpin) is found in the salivary secretome of *Ochlerotatus triseriatus* [58], which indicates its antimicrobial functions. Furthermore, serine/threonine protein kinase may regulate the functions of other proteins by means of phosphorylation, similar to phosphoproteins found in the saliva of *Haemaphysalis longicornis* [59]. N-acetylgalactosaminyltransferase 3, which is associated with carbohydrate binding and distributed in female *Aedes aegypti* [60], was also identified in *C.lividipennis*. Chondroitin proteoglycan-2, which regulates the host *Bactrocera dorsalis* during parasitization with the parasitoid *Diachasmimorpha longicaudata* [61], suggests its potentially crucial role between the two insect species, while the exact function of chondroitin proteoglycan-2 in *C.lividipennis* remains to be further studied. Notably, cathepsin D, an N-/C-terminal TAXI domain-containing protein, known for its inhibitory function against phytopathogens [62], was also found in *C.lividipennis*. We speculate that this protein may assist rice seedlings in resisting pathogens, thereby protecting their food - the insect eggs - from infection, but this hypothesis warrants further investigation.

In the saliva of *A.lucorum*, apolipophorin-III is involved in the induction of host cell death [30]. GSTs play crucial roles in host adaption, for instance, the GST salivary effector Me47 is responsible for modifying plant responses to aphid infestation [63]. Likewise, the mucin-like domain-containing protein is associated with host adaption, for example, the mucin-like protein NlMul in saliva assists *N.lugens* in dealing with plant resistance [2]. Notably, the apolipophorin-III, GST, and mucin-like domain-containing protein are prevalent in various herbivorous insects. On the other hand, the Kazal-like domain-containing protein is related to the breakdown of plant secondary metabolites and associated with xenobiotics detoxification, like insecticides [3, 31]. The up-regulation of a GST and Kazal-like domain-containing protein when feeding on fresh green beans helps to support these speculations. Moreover, venom allergen 5 and pathogenesis-related protein 5 are associated with plant pathogenesis, and pappalysin-1 takes part in the insect immune response [64]. In addition, the functions of 25 *C.lividipennis*-specific and 19 *A.lucorum*-specific salivary proteins, which lack homologs in the eight other species, require further investigations. To sum up, these unique detoxification enzymes may contribute to the distinct feeding behaviors observed in these two mirid bugs.

## Conclusion

According to the findings in this study, two mirid bugs, including the zoophytophagous *C.lividipennis* and the phytozoophagous *A.lucorum*, employ several conserved salivary secretions to shape the overlapping feeding patterns. However, a plethora of unique salivary secretions may drive the evolution of the predominant feeding behavior responsible for their survival. Research from the perspective of saliva not only deepens our understanding of the intricate feeding mechanisms but also lays a foundation for future environmentally friendly pest control by harnessing predatory insects.

## Methods

### Insect rearing

*C.lividipennis* was collected from the experimental field of Jiaxing Academy of Agricultural Sciences, Zhejiang Province. Meanwhile, *A.lucorum* was obtained from the Institute of Plant Protection, Henan Academy of Agricultural Sciences, Henan Province. *C.lividipennis* was reared with *N.lugens* and supplemented with rice seedlings (*Oryza sativa* strain Xiushui) to encourage *N.lugens* oviposition, whereas *A.lucorum* was reared with green beans. The rice seedlings and green beans are widely used varieties in agricultural production and are available for purchase in the market. Both mirid bugs were kept in a phytotron (26.5 ℃, relative humidity 65–75%, and a 14-h/10-h light/dark photoperiod) at Ningbo University, Ningbo, China.

### Survival rate analysis of *C.lividipennis*

The 3-leaf stage rice seedlings were exclusively utilized to explore the impact of rice sap on the survival of *C.lividipennis*. ddH_2_O feeding and no feeding were set as the control conditions. The survival rates were systematically recorded at 12-h intervals, with altogether 10 individuals in each treatment group. Statistical significance for each survival rate was assessed using SPSS 27.0.1 (Chicago, IL, USA), with One-Way ANOVA method.

### RNA-seq analysis

First of all, *C.lividipennis* and *A.lucorum* were anesthetized using carbon dioxide. Subsequently, the SGs, guts, and other carcasses without SGs and guts from both insects were carefully dissected under a microscope (Olympus, Japan). Thereafter, the total RNA was extracted from each sample using the Trizol RNA Isolater Total RNA Extraction Reagent (Vazyme Biotech, Nanjing, China), in line with the manufacturer’s protocol. Afterwards, the RNA concentration, quantity, and integrity were assessed by the Agilent 2100 bioanalyzer (Agilent Technologies, California, USA). Then, to synthesize cDNA, the mRNA was purified from the total RNA using magnetic beads carrying Oligo(dT). The purified cDNA then experienced end repairing, adenylation of 3’ ends, and ligation of adaptors. AMPure XP beads (Beckman Coulter, Beverly, USA) were used to screen out 370–420 bp cDNA. Next, the sequencing library was generated through PCR, and AMPure XP beads were further used in a second purification step. Finally, RNA-seq was conducted on the Illumina Novaseq 6000 platform (Novogene, Tianjin, China). The quality summary regarding library sequencing is listed in (Supplementary Table 8), with over 40 million clean reads being obtained in each library.

### Transcriptome mapping and gene annotation

By adopting Trimmomatic 0.39 [65], low-quality and adapter sequences were filtered out to obtain clean reads. Using HISAT2 2.2.1 and SAMtools 1.6, these clean reads were later mapped to the reference genomes of *C.lividipennis* and *A.lucorum* (Genbank accession numbers: ASM1960339v1 and ASM973950v2, respectively) with the ‘--dta’ parameters. The gene structure of *C.lividipennis* was predicted by Braker 3.0.2 [66] with default parameters within the Singularity 3.10.0 container runtime. The reported official gene set was used for *A.lucorum* [26]. Subsequently, the predicted coding sequences of assembled unigenes were analyzed using the website tool PANNZER (http://ekhidna2.biocenter.helsinki.fi/sanspanz/) to obtain the functional description data. Additionally, a local blast against non-redundant (NR) database on the NCBI website (https://www.ncbi.nlm.nih.gov/), Uniprot, and GO database was performed. GO annotation was performed through the EGGNOG-MAPPER website (http://eggnog-mapper.embl.de/) and TBtool v2.019 software [67]. Finally, gene counts were analyzed using Stringtie 2.2.1 with the “-B -e -o” parameters [68] and the prepDE.py script.

### Tissue-specific expression analysis by qRT-PCR

RNA isolation from the SGs, guts, and carcasses of *C.lividipennis* and *A.lucorum* was performed following the same procedure outlined in the RNA-seq section. Subsequently, 400 ng of RNA was utilized for reverse transcription (RT) to prepare cDNA using the HiScript II Q RT SuperMix (Vazyme, Nanjing, China). For qRT-PCR, the reaction system comprised 5 µl of SYBR Green Supermix Kit (Vazyme, Nanjing, China), 2.4 µl of ddH_2_O, 2 µl of the 20-diluted cDNA, and 0.6 µl of primers (10µM for each primer). qRT-PCR was conducted on the ABI QuantStudio 5 equipment (Thermo Fisher Scientific, USA) with the following cycling conditions: pre-denaturation at 95 °C for 5 min, followed by 40 cycles of denaturation at 95 °C for 10 s, annealing, and sequence extension at 60 °C for 30 s. Housekeeping genes *ClRPS15* [38] and *Alβactin* [69] in *C.lividipennis* and *A.lucorum* served as reference genes, respectively. The relative expression levels of target genes were calculated using the 2^−ΔΔCt^ method [70], with three biological replicates, each comprising three technical repetitions. The primers used for qRT-PCR are listed in (Supplementary Table 9). The presented data were shown as means + SEM. *Statistical analysis was performed using GraphPad Prism 8.0.2 software, with the two-tailed unpaired t-test method.* “ns” denotes *P* ≥ 0.05 (indicating no significant difference), “*”, “**”, and “***” denotes *P* < 0.05, 0.01, and 0.001, respectively (indicating significant, very significant, and extremely significant difference, respectively).

### Prediction of salivary secretions prediction and comparative analysis

By comparing the gene counts in guts and carcasses, the significantly highly expressed genes in SGs were obtained by edgeR 3.18 with the 0.4 variation coefficient. Subsequently, abundantly (top 1000 genes) and significantly expressed genes in SGs were identified. Considering the secretion characteristics of saliva, the SignalP 6.0 website (https://services.healthtech.dtu.dk/services/SignalP-6.0/) was used for predicting signal peptides. At last, the putative salivary secretions from both mirid bugs were cross-referenced using the OrthoFinder program. Afterwards, homologs of the top 1000 genes and putative salivary secretions were searched against the genomes of model species *Drosophila melanogaster*, and Hemiptera species *N.lugens* (Delphacidae), *Acyrthosiphon pisum* (Aphididae), *B.tabaci* (Aleyrodidae), *Riptortus pedestris* (Coreidae), *Halyomorpha halys* (Pentatomidae), as well as two insects belonging to the family Miridae, *Nesidiocoris tenuis* and *L.lineolaris*, using TBtool v2.019 software (Genbank accession numbers: GCF_000001215.4, PRJNA669454, GCF_005508785.2, GCF_001854935.1, GCA_019009955.1, GCF_000696795.2, GCA_902806785.1, and GCA_030264115.1, respectively).

### Function analyses of 10 salivary protein-coding genes

The female 5th instar *C.lividipennis* individuals were divided into two groups, with one group being reared with *N.lugens* nymphs and eggs, while the other group reared with fresh rice seedlings. Similarly, the female 5th instar *A.lucorum* individuals were divided into two groups, including one group being reared with eggs of *H.armigera*, while the other group reared with fresh green beans. Four days later, the total RNAs from each *C.lividipennis* and *A.lucorum* individual were extracted following the protocols outlined in the RNA-seq section. Subsequently, the expression levels of 10 salivary protein-coding genes were assessed by qRT-PCR, using the same procedures and statistical analysis detailed in the Tissue-specific expression analysis by qRT-PCR section. The primers used for qRT-PCR are listed in (Supplementary Table 9).

### Electronic supplementary material

Below is the link to the electronic supplementary material.

Supplementary Material 1

Supplementary Material 2

## Data Availability

All raw sequence data were submitted to the Genome Sequence Archive in the National Genomics Data Center, China National Center for Bioinformation/Beijing Institute of Genomics, Chinese Academy of Sciences (https://ngdc.cncb.ac.cn/gsub/), with the GSA CRA011118 under Bioproject PRJCA017140.
