# Supplementary material for: Comparative transcriptomic analysis of salivary glands between the zoophytophagous Cyrtorhinus lividipennis and the phytozoophagous Apolygus lucorum
Source: BMC Genomics. 2024 Jan 11;25:53. doi: 10.1186/s12864-023-09956-4 (PMC10785411; doi:10.1186/s12864-023-09956-4)
Supplement: Supplementary file 1 — Supplementary Material 1 [file 12864_2023_9956_MOESM1_ESM.docx]

**Supplementary Figures**


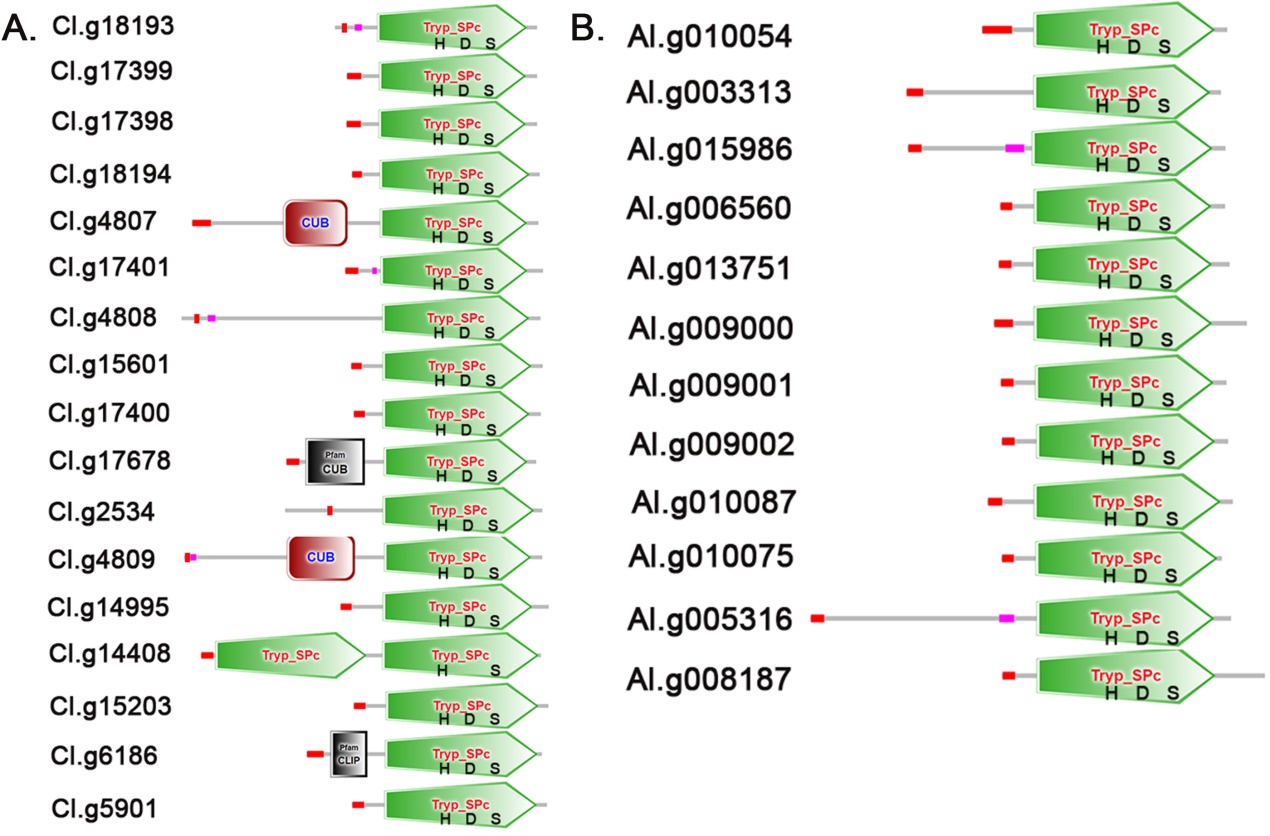


**Supplementary Fig. 1 Signal peptide and domain analysis of the predicted serine proteases of *C.lividipennis* (A) and *A.lucorum* (B)**

Red: A signal peptide indicated with SignalP 6.0 website; green: a typical serine protease domain (Tryp_SPc); H, D, S: His-Asp-Ser.


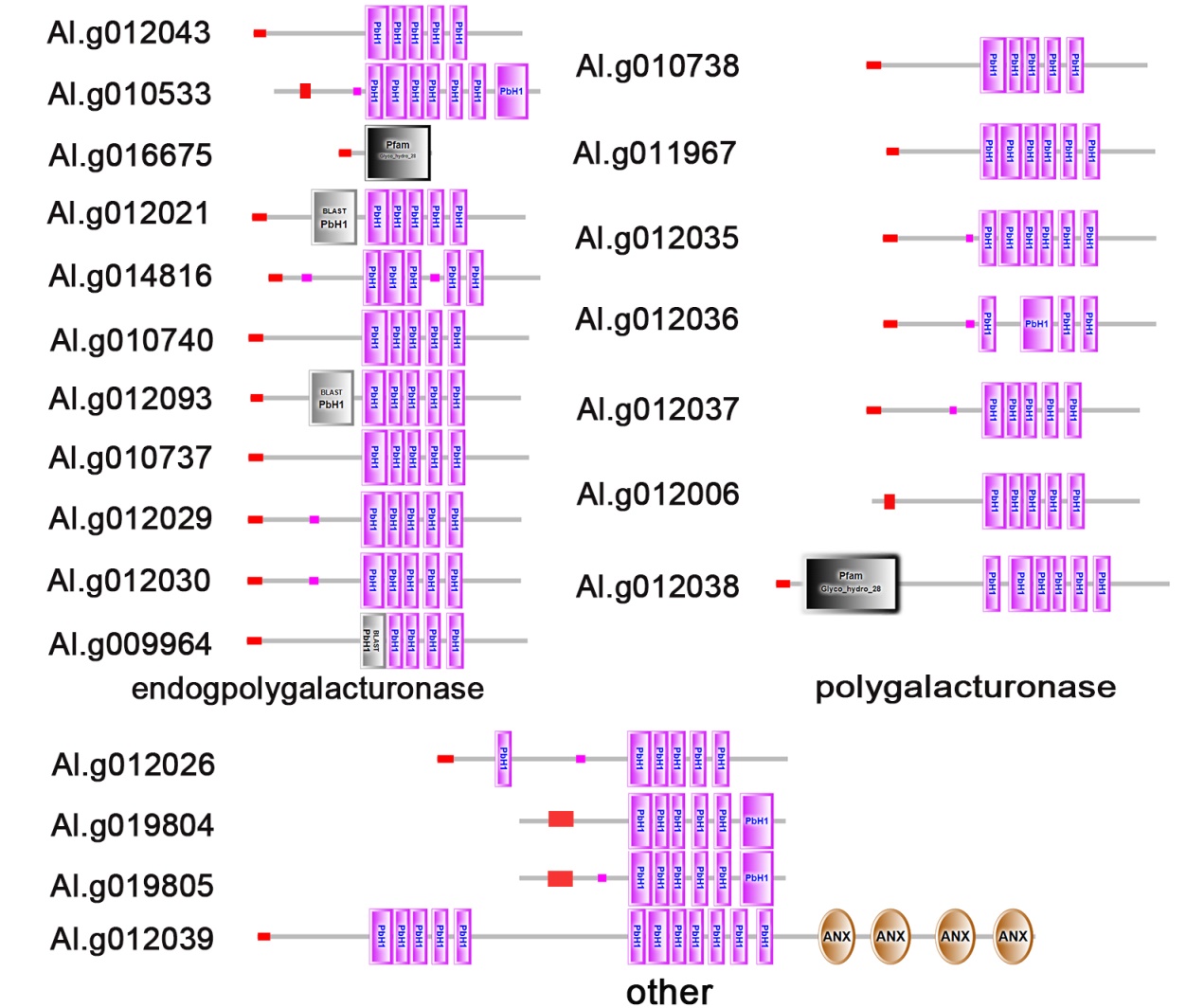


**Supplementary Fig. 2 Signal peptide and domain analysis of the predicted polygalacturonases of *C.lividipennis* (A) and *A.lucorum* (B)**

Red: A signal peptide indicated with SignalP 6.0 website; Purple: Glyco_hydro_28 domains.
